# GREG—studying transcriptional regulation using integrative graph databases

**DOI:** 10.1093/database/baz162

**Published:** 2020-02-13

**Authors:** Songqing Mei, Xiaowei Huang, Chengshu Xie, Antonio Mora

**Affiliations:** 1 School of Basic Medical Sciences, Guangzhou Medical University, Panyu Campus of Guangzhou Medical University, Xinzao, 511436 Guangzhou, P.R. China; 2 Joint School of Life Sciences, Guangzhou Medical University and Guangzhou Institutes of Biomedicine and Health (Chinese Academy of Sciences), Panyu Campus of Guangzhou Medical University, Xinzao, 511436 Guangzhou, P.R. China

## Abstract

A gene regulatory process is the result of the concerted action of transcription factors, co-factors, regulatory non-coding RNAs (ncRNAs) and chromatin interactions. Therefore, the combination of protein–DNA, protein–protein, ncRNA–DNA, ncRNA–protein and DNA–DNA data in a single graph database offers new possibilities regarding generation of biological hypotheses. GREG (The Gene Regulation Graph Database) is an integrative database and web resource that allows the user to visualize and explore the network of all above-mentioned interactions for a query transcription factor, long non-coding RNA, genomic range or DNA annotation, as well as extracting node and interaction information, identifying connected nodes and performing advanced graphical queries directly on the regulatory network, in a simple and efficient way. In this article, we introduce GREG together with some application examples (including exploratory research of Nanog’s regulatory landscape and the etiology of chronic obstructive pulmonary disease), which we use as a demonstration of the advantages of using graph databases in biomedical research.

**Database URL:** https://mora-lab.github.io/projects/greg.html, www.moralab.science/GREG/

## Introduction

The study of a regulatory process is a combination of the concerted action of transcription factors (protein–DNA binding data), protein complexes and co-factors (protein–protein interaction data) and regulatory non-coding RNAs (ncRNAs) (ncRNA–DNA binding and ncRNA–protein interaction data), as well as chromatin interactions (DNA–DNA interaction data). Therefore, the combination of the above five types of data in a single repository offers new possibilities regarding generation of biological hypotheses, such as co-regulation, transcription factor (TF) multimerization, enhancer–promoter interactions, protein–ncRNA complex structures and the role of 3D organization in gene regulation, as well as mechanisms combining all the previous evidence in a complex regulatory landscape.

We introduce GREG (The Gene Regulation Graph Database) (1), an integrative database and web resource that we have developed to offer an integrative analysis of transcriptional regulation in a simple graphical way. GREG is not only a database but also a visualization and data exploration platform. GREG’s web platform allows genomic researchers to (i) visualize all the known interactions between proteins, RNAs and DNA for a query transcription factor, RNA or genomic range of interest; (ii) directly extract node and interaction information (such as data source, experimental methods and other details) from the resulting integrative network; (iii) expand the queried graph and explore it by merely clicking on nodes and edges; and (iv) perform advanced graphical queries such as finding the shortest paths that link two biomolecules in a cell’s regulatory landscape. In this paper, we describe GREG’s structure and share some examples applied to the exploratory research of regulatory landscapes and the etiology of chronic obstructive pulmonary disease (COPD).

## Materials and methods

Our approach is based on building a ‘graph database‘ with interaction information coming from multiple source databases: 4DGenome (2) for DNA–DNA interactions, iRefIndex (3) for protein–protein interactions, Cistrome (4) for protein–DNA binding, LnChrom (5) for long non-coding RNA (lncRNA)–DNA interactions, starBase (6) for lncRNA–protein interactions and Gencode (7) for DNA annotation. Graph databases are an approach that has already been adopted for other bioinformatics projects such as Bio4j (8), cyNeo4j (9), BioGraph (10), MELODI (11) and Reactome (12).

Graph databases are able to model complex relationships that are difficult to model using relational databases. Besides that, they allow us to observe such relationships directly and get their associated data from hovering over nodes and edges instead of accessing and browsing tables. In addition, they allow us to build complex graphical queries such as those based on shortest paths, clustering and network expansion. GREG’s database is written in Neo4j (13), while its web platform is written in Java (14), JavaScript (15) and vis.js (16).

GREG is also a data integration project, the main challenge being the need to integrate protein and lncRNA data (which are biomolecules) with genomic coordinates (which are numerical ranges). To accomplish that, all genomic DNA has been binned. A bin is an arbitrary segment of a chromosome with a pre-defined size, which is more susceptible to be added to a graph than a single base pair and therefore is useful to organize heterogeneous interaction information such as that from proteins and DNA. Currently, GREG works with bins of a fixed size. Users can choose between large bins (200 kb) or small bins (2 kb). While several tools produce networks of gene–gene relationships, and a few resources can be found for enhancer–promoter interactions, GREG includes interaction information for every single DNA bin in the genome.

Another challenge was the harmonization of bins and small protein-binding sites with data coming from chromatin interaction technologies of vastly different resolutions, which GREG tackles by introducing special ‘chromatin range’ nodes that define chromatin interactions. The chromatin range is a specific range, as reported in a chromatin interaction experiment. Therefore, chromatin ranges are the nodes linking chromatin–chromatin interactions, and they could be larger or smaller than DNA bins, depending on the chosen size of the bin and the resolution of the chromatin interaction experiment.

**Figure 1:**
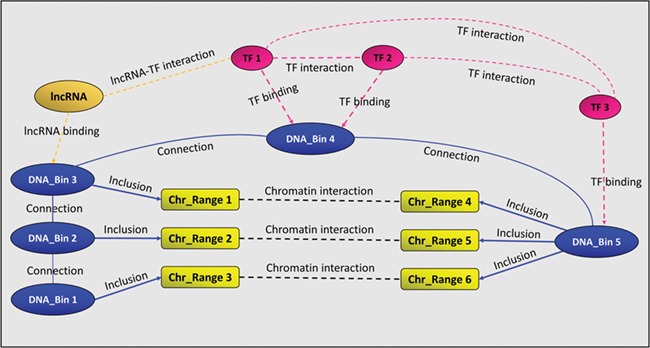
A GREG regulatory landscape. A regulatory landscape contains DNA bins (blue), which are ‘connected’ between them. DNA-binding proteins or TF (red) ‘bind’ the DNA bins and ‘interact’ with other proteins (red). DNA bins (blue) also ‘include’ some DNA ranges (yellow), which, in time, ‘interact’ with other ranges through chromatin–chromatin interactions (black). lncRNAs (orange) can interact with both bins and proteins.

In addition to the above-mentioned binding and interaction relationships, GREG stores two auxiliary relationships called ‘connection’ and ‘inclusion’. The first one links consecutive DNA bins, in order to keep chromosomes together, while the second one links chromatin interaction ranges to DNA bins, as explained above. Figure 1 summarizes GREG’s data model.

Nodes and relationships contain additional information stored as properties. In GREG, such properties can be accessed by hovering over nodes and relationships. Table 1 summarizes the properties included in GREG v.1.0.

**Table 1:** Properties of all nodes and relationships in GREG

**Node or relationship**	**Properties**
DNA bin	**Name:** bin ID
	**Details:** GENCODE information, including genes in the bin
	**Start:** genomic coordinates of the bin’s starting point
	**End:** genomic coordinates of the bin’s ending point
TF	**Name:** gene symbol of the DNA-binding protein (note: TF nodes are mainly transcription factors, but GREG also includes chromatin remodeling proteins, histone variants and other types of DNA-binding proteins)
Chromatin range	**Start:** genomic coordinates of the range’s starting point
	**End:** genomic coordinates of the range’s ending point
lncRNA	**Name:** lncRNA symbol
TF–TF interaction	**UIDA:** iRefIndex’s identifier of interactor A
	**UIDB:** iRefIndex’s identifier of interactor B
	**Method:** experimental method
	**Icrigid:** iRefIndex’s identifier of the canonical version of the interaction
	**Edgetype:** iRefIndex’s code for interaction type (X for binary relationships, C for complexes, Y for polymers)
	**Pubmeds:** PubMed IDs describing the interaction
	**Confidence:** iRefIndex’s bibliometric indexes of confidence (np, lpr, hpr)
TF–DNA binding	**GEO:** Gene Expression Omnibus ID for the dataset containing the binding relationship
	**SourceDB:** source database (currently only CistromeDB)
	**OtherGEO:** other GEO IDs
	**CellType:** cell type
	**Start:** genomic coordinates of the binding site’s starting point
	**End:** genomic coordinates of the binding site’s ending point
	**Confidence:** Cistrome’s *q*-value of the binding relationship
DNA–DNA interaction	**SourceDB:** source database (currently only 4DGenome)
	**Method:** experimental method
	**PubMedID:** PubMed ID describing the interaction
	**CellType:** cell type
	**Confidence:** confidence score from the original study, according to 4DGenome
lncRNA–DNA interaction	**LncRNA_ID:** lncRNA ID
	**SourceDB:** source database (currently only LnChrom)
	**Method:** experimental method
	**Genomic_region:** genomic coordinates of the binding site
	**Confidence:** low- or high-throughput study
	**PubMedID:** PubMed ID
	**CellType:** cell type

GREG v.1.0 consolidates data from eight human cell types, including three stem cells (H1, IPS19.11, IPS6.9), four cancer cells (A549, K562, MCF-7, HeLa) and one other cell line (IMR-90), from the following source databases: iRefIndex (v.13.0), Cistrome (v.2018), Gencode (release 21), 4DGenome (downloaded June 2018), LnChrom (downloaded June 2018) and starBase (downloaded June 2018). iRefIndex, in turn, consolidates data from multiple interaction databases, including BIND, BioGRID, CORUM, DIP, HPRD, InnateDB, IntAct, MatrixDB, MINT, MPact, MPIDB and MPPI. The databases were chosen according to different criteria including openness, comprehensiveness and popularity. The cell types were chosen because they can be found in all the selected databases; i.e. there is available information on each of the types of interactions under study. All genomic data were converted to hg38 human genome using University of California Santa Cruz’s liftOver. All downloaded files were processed using R and then merged into a Neo4j graph using a script written in Cypher (Neo4j’s query language) through Python’s Py2neo library (17). The corresponding integration scripts are available at https://github.com/mora-lab/GREG. The database was built using Python 3.6, Py2neo v4 and Neo4j Community Edition 3.5, while the web platform was built using Java 1.8.0 and vis.js community edition.

## Results

### Description

**Figure 2:**
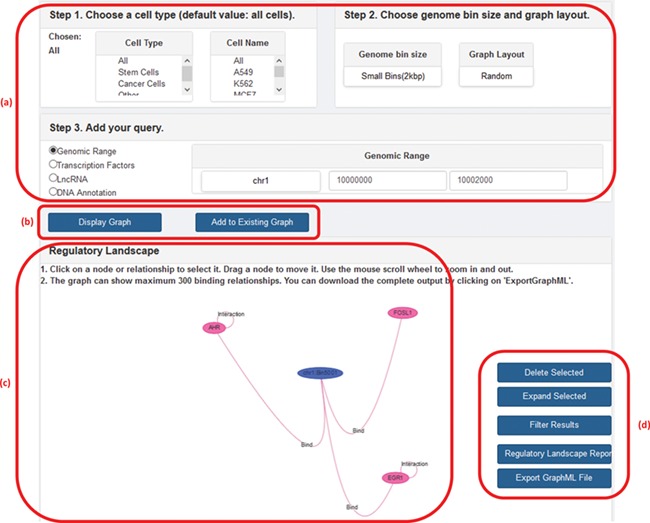
A screenshot of GREG’s web platform (basic mode). (a) Input data: cell type, DNA bin size and query (genomic range, transcription factor, lncRNA or DNA annotation). (b) Options to run query: either display new graph or add query to existing graph. (c) Results window: data associated with each node or relationship (see Table 1) can be visualized by hovering over the graph. (d) Options to manage results: delete or expand selected nodes or relationships, filter results by a given type of relationship, print a summary report or export a graph to a graph format.

GREG not only can be accessed directly as a Neo4j database using Cypher but also can be accessed from our website (1). In the web platform, the user can specify a cell type, chromosome and DNA bin size and ask for the network of interactions around a given TF, lncRNA, genomic range or a specific DNA annotation (gene name or gene ID). The output, or ‘regulatory landscape‘, will be the sub-graph induced by the query (i.e. the sub-network generated by all protein–protein, protein–DNA, protein–lncRNA, lncRNA–DNA and DNA–DNA interactions according to the query), and it can be visualized on the screen or be downloaded in a graphical format such as graphML (18) for further graphical processing. Other features of the web version include choosing between random or hierarchical network layouts, filtering a regulatory landscape according to user-defined relationships, possibility of moving nodes around the network, expanding a node (in terms of a specific relationship), deleting a node or an interaction, publishing a text report with summary statistics of the input and output, and the possibility of adding a second search on top of the first one. Figure 2 shows a screenshot of GREG’s web platform (basic mode).

The website also contains a menu for advanced options, which essentially includes the most typical network analysis operations, starting with shortest-path computation. When using ‘shortest paths’, it is possible to find if two nodes are connected or if their relationship is mediated by a third node in the integrative network, which could suggest biological mechanisms. Such connectivity analyses may be complementary to the usual correlation analyses. More details about GREG’s implementation and several frequently asked questions can be found on the website (1).

Currently, GREG’s main limitation is that search time grows with the size of the genomic range. That means that it is quite fast for searches on molecules (proteins, lncRNAs) or small genomic regions, while it slows down for searches on very large genomic regions such as whole-genome or whole-chromosome tasks, which is the cost we pay for including all non-coding regions. Getting access to GREG using Neo4j/Cypher allows the user to perform any type of Cypher query in the database beyond the ones included in our web platform; in order to do that, the user must contact us and wait for us to send instructions together with a temporary username and password. Unless the user has experience with Neo4j and Cypher, we recommend using the web platform here described, which does not require any login credentials.

When using small bins, the consolidated GREG network consists of 2 778 332 nodes and 19 384 819 relationships. Online supplementary material Table S1 shows that most of the nodes correspond to chromatin interaction ranges and DNA bins. Accordingly, online supplementary material Table S2 shows that most of the relationships in the GREG network correspond to protein–DNA binding relationships, followed by DNA–DNA interaction relationships. Besides statistics of nodes and relationships, we have also characterized GREG in terms of the network structure. Online supplementary material Figure S1 shows the degree distribution of one chosen chromosome (chr12), revealing the existence of many nodes with a low degree and a few hub nodes with a higher degree. We performed additional analyses of the hub nodes with the highest degree in chr12, which we report in online supplementary material Figure S2. Gene set analysis shows that some of such hubs are functionally enriched; for example, Bin35 (the fifth largest DNA hub in GREG’s chr12) contains 28 genes, which are enriched on glycolysis and gluconeogenesis genes. Another descriptor of network structure is the existence of modules or groups of nodes that are densely connected internally. Modules in protein interaction networks can be indicative of protein complexes, while modules of chromatin interaction networks can be indicative of topologically associated domains (TADs). We build a higher-order type of modules from GREG by including all available relationships and use them to describe a chromosome or the entire genome. Online supplementary material Figure S3 shows all the modules (communities) involving chr12 bins (50 modules in total) together with the number of bins per module. According to the clusterProfiler R package (19), all of those modules show enrichment in at least one Kyoto Encyclopedia of Genes and Genomes pathway or Gene Ontology term.

### Example 1—exploring Nanog’s regulatory landscape

We will illustrate the advantages of using GREG through the study of the regulatory landscape of the human Nanog locus. NANOG is a transcription factor involved in embryonic stem cell proliferation, renewal and pluripotency. First, we will solve simple questions involving a single type of biological interaction, which is a task that can also be done using other resources. Then, we will proceed to more complex scenarios involving multiple types of relationships.

The first question we try to answer is if the NANOG protein is acting as a multimer. Following the procedure in online supplementary material Example 2.1, we find that NANOG shows a self-interaction edge, which indicates that, indeed, NANOG interacts with itself. If we check the properties of that relationship, we can find the identifier of the interaction, which we can use in iRefR (20) or iRefWeb (21) if we are interested in more details. Indeed, it has been known for a long time that NANOG functions as a dimer (22). The second simple question is which TFs are regulating the Nanog gene expression. Following the procedure in online supplementary material Example 2.2, the resulting regulatory landscape shows us a complex multitude of protein and chromatin interactions that summarizes the existing knowledge on Nanog’s regulation. We can select ‘Regulatory Landscape Report‘ and see that the NANOG gene spans 7 GREG DNA bins bound for 42 TFs and including 13 DNA ranges that interact with other DNA ranges. Some of the proteins include POU5F1, EP300, CTCF, POLR2A, H2AZ, RAD21, YY1 and NANOG itself. The third simple question is whether the Nanog gene is rich in chromatin interactions. Following the procedure in online supplementary material Example 2.3, we obtain a network including 16 DNA–DNA interactions; we can find additional details in the corresponding report.

GREG is more valuable for studying more complex scenarios. The fourth problem we exemplify is as follows: we know that there is an enhancer 45 kb upstream Nanog that regulates both Nanog and Dppa3 in mouse embryonic stem cells (23). Is that enhancer active in human K562 cells? Following the procedure in online supplementary material Example 2.4, we can observe that none of the resulting 16 DNA ranges is located around 45 kb upstream Nanog. Therefore, there is no evidence of that enhancer being active in human K562 cells. The fifth question involves the characterization of the ‘topologically associated domains‘, or TADs, which are those DNA segments highly enriched in chromatin–chromatin interactions. Following the procedure in online supplementary material Example 2.5, we obtain a dense network or ‘chromatin hub‘. In the report, we find that such a hub includes 101 chromatin interactions, 1 lncRNA–DNA binding, 2931 TF–DNA binding sites and 1097 protein–protein interactions, which suggests that this TAD might be important in terms of DNA’s 3D organization and gene regulation.

### Example 2—exploring genomic mechanisms of COPD

COPD is a chronic respiratory disease that consists of progressive airflow limitation and inflammatory response of the airways and lungs. COPD-related injuries may occur to both tissue and the extra-cellular matrix (ECM) through processes such as ECM proteolysis, apoptosis and oxidative stress. Other COPD cases are related to problems with self-repair mechanisms. It is also known that epigenetic changes and cell senescence can both increase inflammation and decrease tissue repair (24). Genetic association studies suggest that the best candidate genes for COPD are CHRNA3, CHRNA5, IREB2, HHIP, FAM13A and AGER [25]. Other candidates include SERPINA1, TGFB2, MMP12 and RIN3 (25), as well as IL13, MMP9, SOD3 and TGFB1 (26). However, the mechanisms in which such genes affect COPD risk are not well known. Online supplementary material Table S3 summarizes relevant information regarding those genes.

We have used GREG to shed light on the mechanisms that could link COPD’s candidate genes to the phenotype. We have collected the genomic coordinates of the main single-nucleotide polymorphism (SNPs) related to COPD according to SNPedia (26) (online supplementary material Table S4); then. we have chosen IMR90 cells (corresponding to lung fibroblasts) and searched for the proteins, lncRNAs and genomic regions that may be interacting with the bins around such SNPs.

As a first result, the lung fibroblast’s GREG network shows that several SNP-containing COPD-associated genes form chromatin hubs with other genes in the same chromosome (see online supplementary material Table S5). Based on such information, GREG allows us to hypothesize that mutations in CHRNA3, IL13 and MMP9 genes may respectively affect chromatin hubs in chr15 (genes associated with effects of smoking), chr5 (genes associated with cytokine signaling, cell cycle, transport and senescence) and chr20 (genes associated with immunity, inflammation and transport). These are all processes that have been related to COPD in previous transcriptomic and epigenomic studies, but there is no experimental evidence of the existence of such chromatin hubs. In addition, we collected differential expression data for the interactor genes and found that several of them appear to be downregulated in COPD (online supplementary material Table S5), suggesting that some of those DNA interactions may have an effect on gene expression.

Regarding the protein–DNA interactions found by GREG, results are summarized in online supplementary material Table S6. The list of binding nuclear proteins is enriched on several proteins related to DNA interactions and chromatin organization such as CTCF and RAD21, as well as transcription-related proteins such as POLR2A and H2AZ. However, it calls our attention the enrichment on LMNB1 and RB1/RBL1, which are lamina-associated proteins. LMNB1 encodes Lamin B1, which is a component of the nuclear lamina. The role of Lamin B1 in lung cancer has been previously explored (27), and it has also been reported that LMNB1 is downregulated during senescence in IMR90 cells (28). Very recently, Lamin B1 downregulation has also been associated with cellular senescence during COPD (29), although we are not aware of any links between such reports and the candidate COPD genes. RB1 encodes the RB transcriptional co-repressor 1, which is mainly related to the cell cycle and different types of cancer, besides heterochromatin formation, cellular senescence and DNA damage response. It also associates with the nuclear lamina, and it has been suggested that failure to associate with the lamina leads to downstream effects affecting its cell cycle and tumor suppression roles (30). RBL1 has a similar sequence to RB1, and it is therefore believed to have similar functions. Together, GREG’s results point to an enrichment on lamina-related proteins whose function may be affected by COPD-associated mutations. A supporting argument is that cross-talk has been previously reported between three senescence mechanisms: DNA damage, oxidative stress and nuclear shape alterations (31). As the two first mechanisms have been extensively discussed in the context of COPD, the third one becomes plausible.

In summary, GREG’s data integration helps to generate at least two hypotheses regarding the etiology of the disease: one is the existence of specialized chromatin hubs involving COPD genes, with a potential effect on transcription of a second group of genes (which are also present in known COPD pathways). The other one is the possibility of affecting binding of lamina-associated proteins and, therefore, lamina-associated regulation of the COPD genes. GREG’s regulatory landscape has facilitated the generation of a model that combines well-known mechanisms with new reasonable hypotheses that may become the starting point of deeper studies.

## Discussion

The efficiency of biological graph databases has been measured before. Have and Jensen have shown some pros and cons of using a graph database (Neo4j) versus using a relational database (PostgreSQL) for working with the human interaction network generated by the STRING database (consisting of 20 140 nodes and 2.2 million relationships) (32). They concluded that graph databases offer better speeds than relational databases in several specific types of queries: Neo4j happened to be 36 times faster than PostgreSQL in finding the neighbor network, 981 times faster in finding the best-scoring path and 2441 times faster in finding the shortest path. Wiese *et al.* (33) built a gene regulatory graph database and found scalability of query execution using a small and a large Neo4j database. Increasing the number of nodes did not impact the runtime in a significant way while increasing the number of relationships did. However, Neo4j keeps a node/relationship cache, and such cache has a positive effect on runtime. After warming up the cache (34), the execution time for processing queries decreased by 64% compared to the cold-boot (no-caching) scenario, for both a small and a large database, with the execution times being similar between the small and the large datasets.

In addition to efficiency, we suggest four main reasons for using GREG in biological research:

1. Traditional gene regulation analyses become much simpler, faster and powerful using the unified view of GREG’s web platform instead of combining information from multiple other tools, such as genome browsers and the websites of individual interaction databases.
2. The users do not need to get a text or image file as a result and then convert it to a graph. They get the graph instead.
3. There is a possibility of graphical queries, such as extending the query to the neighbors of the query nodes or finding the shortest paths between two genomic regions. Neo4j has multiple methods for community, centrality and shortest-path detection. That means that we can also ask questions such as ‘what happens in the neighborhood of that module/node?', ‘what is the most important node?’ or ‘how are these two regions/nodes connected?'
4. All of these can be accomplished via an intuitive web interface, which requires minimal input from the user, skipping the need to get data from different resources, load it to R or similar computational software, write scripts for data integration and make network analysis with a specific network library.

We have shown that the complex networks retrieved by GREG are useful for exploratory research of the multiple types of interactions around a target molecule or genomic range, as well as in finding potential mechanisms of disease. We can also foresee additional applications in medical and pharmacological research. For example, in the field of disease markers, where research has moved from identifying disease-associated molecules to disease-associated modules on networks (network biomarkers), an approach that may benefit from GREG’s integrative nature. Or in the drug target prediction field, where the multiple types of interactions in a GREG landscape allow a more accurate representation and might improve the performance of network-based drug target identification methods. Besides that, we expect GREG to keep evolving, given that its ‘graph database’ and ‘integrative network’ nature has the advantage of facilitating the addition of more databases, more cell types and more types of network analyses.

## Supplementary Material

Supplementary_File_GREG_19-12-06_baz162
