# Supplementary material for: GREG—studying transcriptional regulation using integrative graph databases
Source: Database (Oxford). 2020 Feb 13;2020:baz162. doi: 10.1093/database/baz162 (PMC7018612; doi:10.1093/database/baz162)
Supplement: Supplementary_File_GREG_19-12-06_baz162 [file supplementary_file_greg_19-12-06_baz162.docx]

**Supplementary file**

**“GREG -Studying transcriptional regulation using integrative graph databases”**

Songqing Mei^1^, Xiaowei Huang^2^, Chengshu Xie^2^, and Antonio Mora^2*^

^1^ School of Basic Medical Sciences, Guangzhou Medical University, Guangzhou (China)

^2^ Joint School of Life Sciences, Guangzhou Medical University and Guangzhou Institutes of Biomedicine and Health (Chinese Academy of Sciences), Guangzhou (China)

^*^ Corresponding author

**Content**

1. Network statistics of GREG v.1.0

2. Example –Exploring Nanog’s regulatory landscape

3. Example –Exploring genomic mechanisms of COPD

**1. Network statistics of GREG v.1.0**

We have generated descriptive statistics of GREG’s database in terms of nodes and relationships, as well as in terms of network structure. Regarding nodes, table S1 shows that GREG’s nodes are mainly chromatin interaction ranges while the number of protein and lncRNA nodes is much smaller. In terms of DNA bins, the large-bin network encapsulates the human genome in approximately 15,000 bins, while the small-bin network has a resolution 100 times higher.

**Table S1.** Number of each type of nodes in GREG v.1.0.

| **Type of nodes** | **Nodes in small-bins network** | **Nodes in large-bins network** |
| --- | --- | --- |
| Protein | 261 | 261 |
| lncRNA | 35 | 35 |
| DNA bin | 1,543,513 | 15,448 (range: from 1,245 bins for chr1 to 234 bins in chr21) |
| Chromatin interaction range | 1,234,523 | 1,234,523 (range: from 127,334 in chr1 to 169 in chrY) |
| Total | 2,778,332 | 1,250,267 |

Regarding relationships, table S2 shows that GREG’s relationships are mainly protein-DNA binding relationships, followed by DNA-DNA interactions, which confirms that most of the database content is related to DNA and not to proteins or lncRNAs.

**Table S2.** Number of each type of relationship in GREG v.1.0.

| **Type of relationship** | **Relationships in small-bins network** | **Relationships in large-bins network** |
| --- | --- | --- |
| Protein-protein interactions | 2,912 | 2,912 |
| Protein-DNA binding | 17,661,016 | 2,358,049 |
| DNA-DNA interactions | 1,672,998 | 1,672,998 |
| lncRNA binding | 47,893 | 14,037 |
| Total | 19,384,819 | 4,047,996 |

Figure S1 shows the degree distribution of the network using large bins (we have selected one chromosome (chr12) for this study). Protein nodes are visibly more connected than DNA nodes, and they have different degree distributions: Most DNA bin nodes have a small degree, while a few nodes have high degrees; in contrast, protein nodes show a more random distribution. For GREG v.1.0, DNA bin nodes show a median degree value of 219 and a maximum value equal to 673, while TF nodes show a median degree of 7,033 and a maximum value of 14,480.


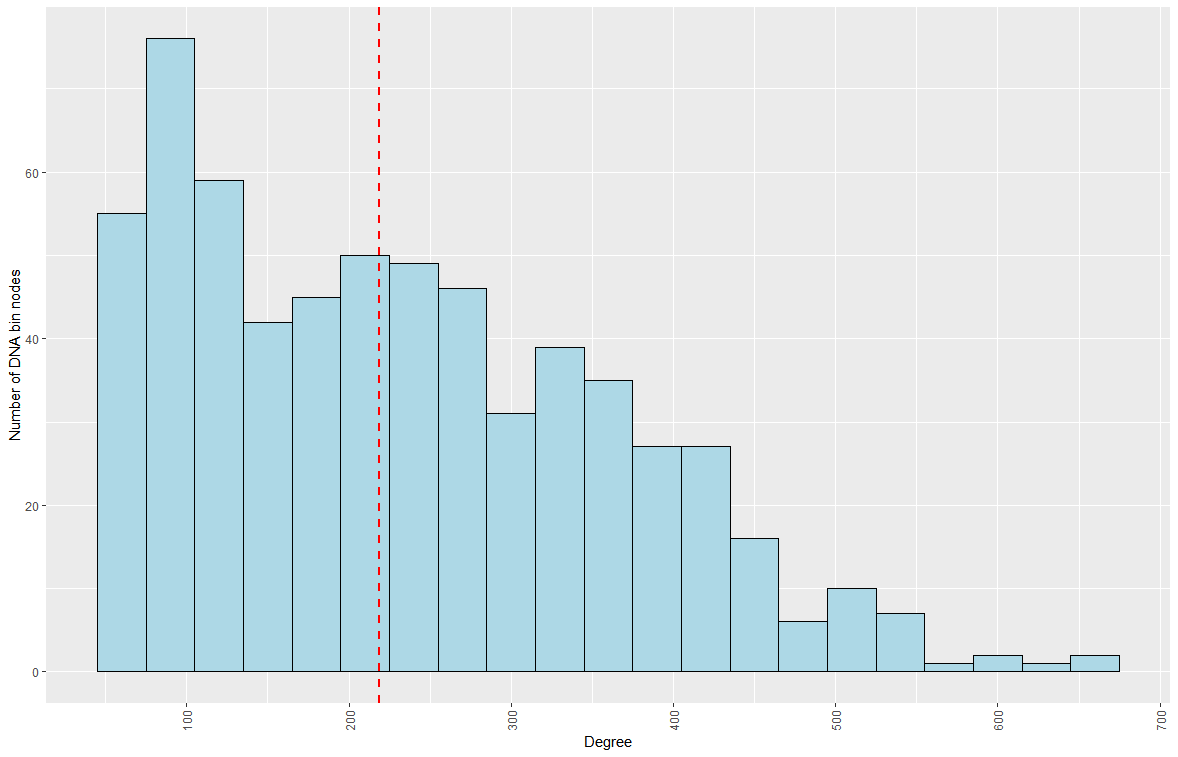


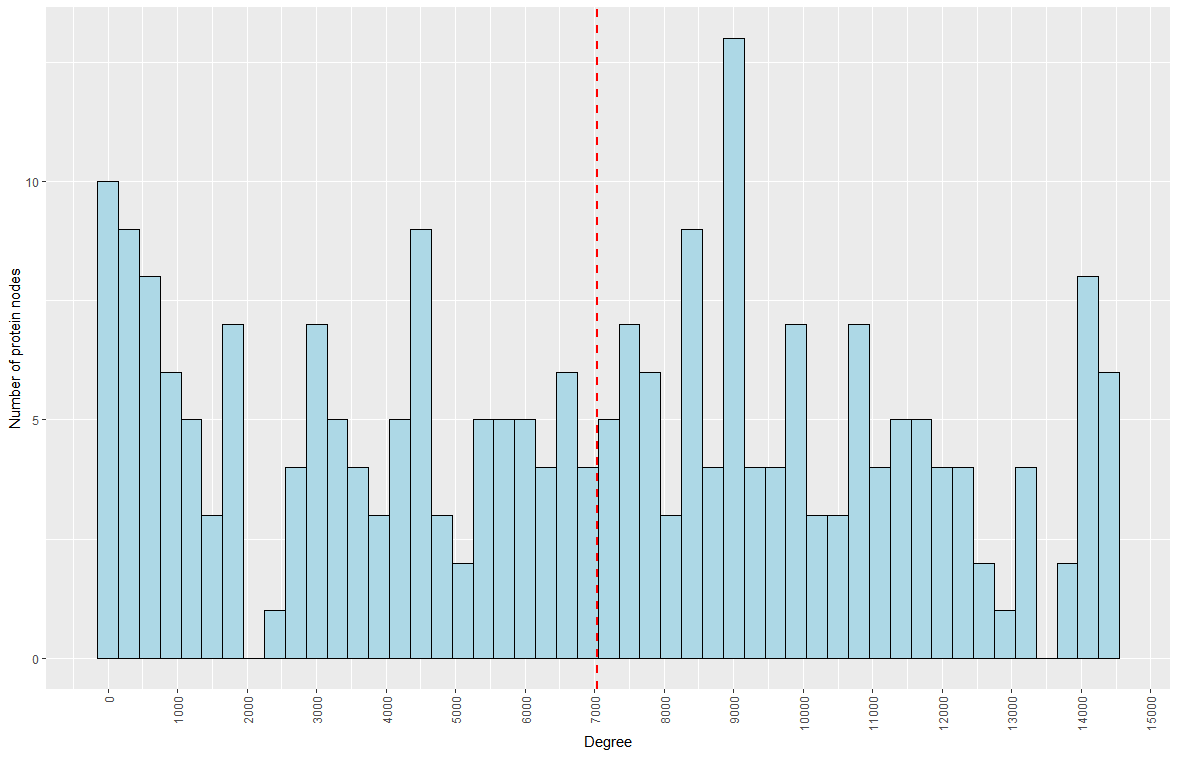


**Figure S1.** Degree distribution of nodes for chr12.

Figure S2 shows the top 20 most connected hubs from the previous distribution. We performed Gene Set Analysis on those hubs and found functional enrichment on many of them. As an example, Bin35 contains 28 genes, which are enriched on glycolysis and gluconeogenesis genes.


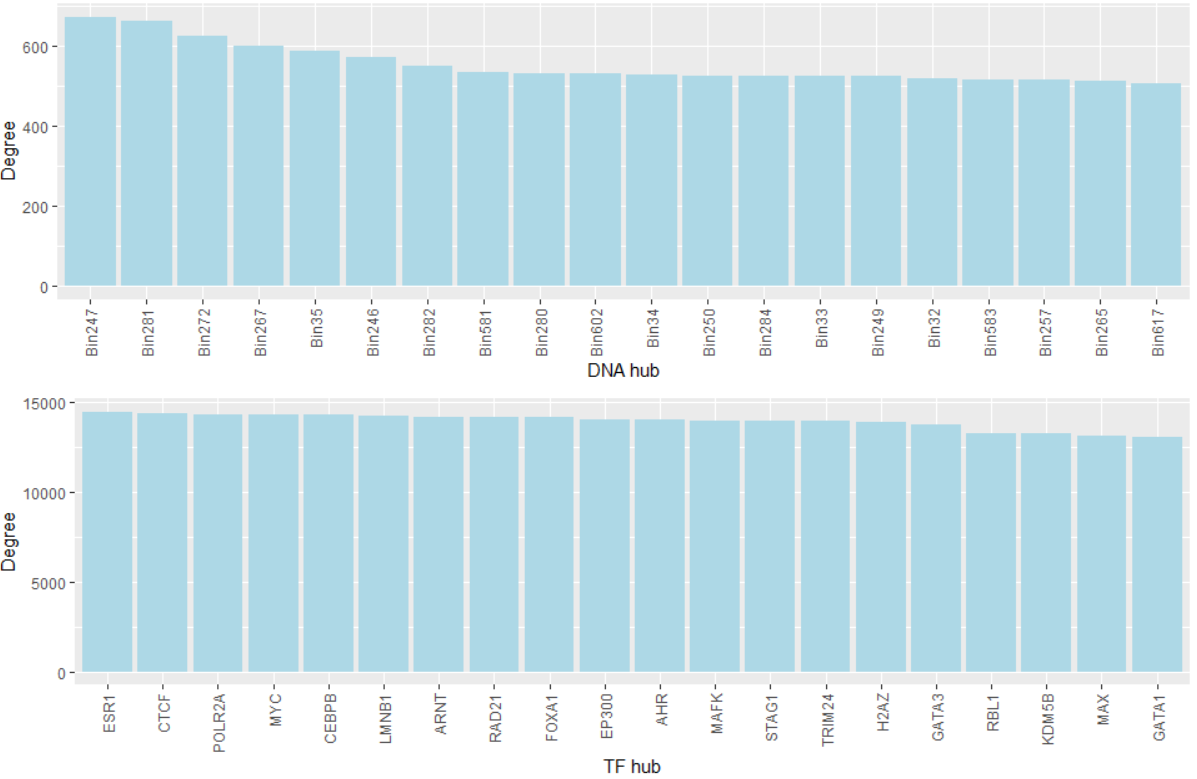


**Figure S2.** Top 20 hub nodes for chr12.

Regarding modules, figure S3 summarizes the size of all modules found in chr12 using large bins. The histogram reveals the existence of a modular organization, with multiple modules of similar size and the existence of one giant connected component where numerous interactions take place. Modules were computed using Neo4j’s Louvain algorithm.


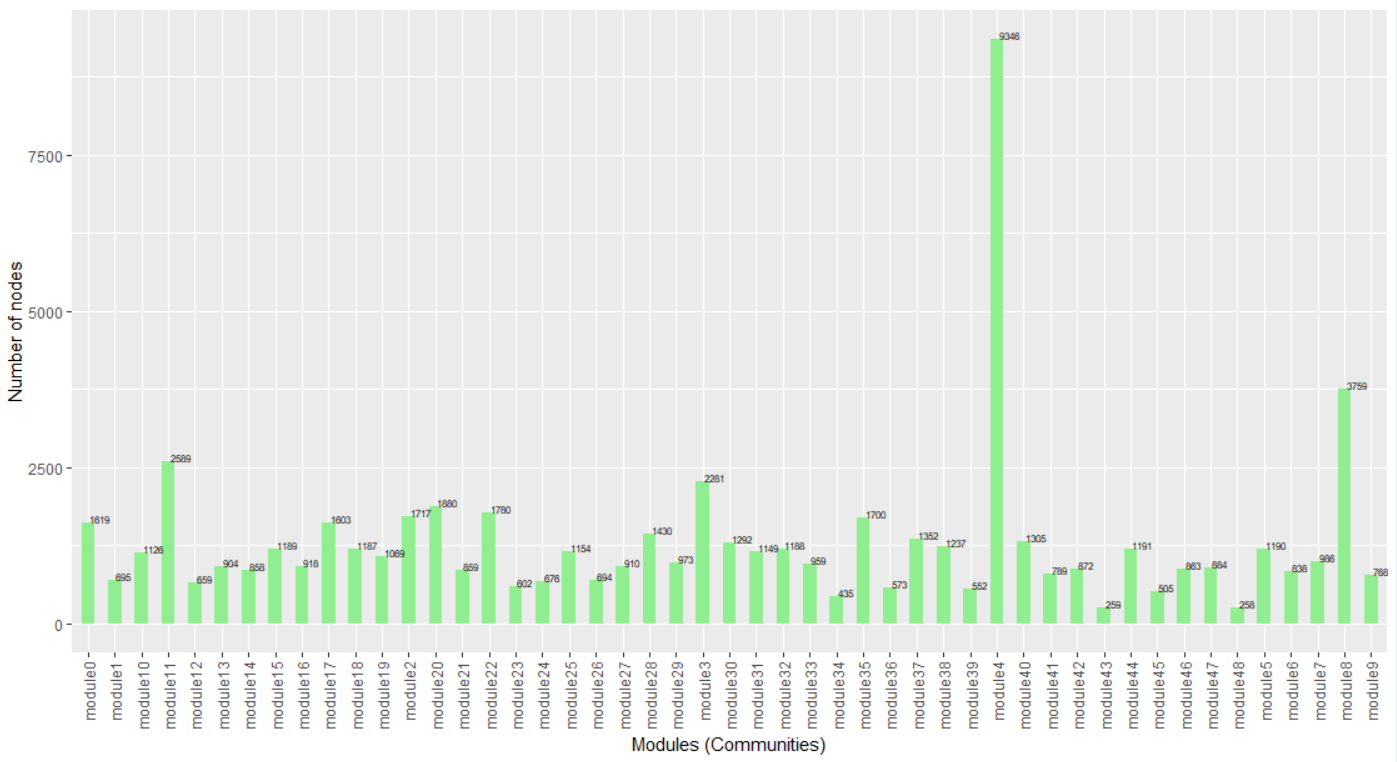


**Figure S3.** All modules from GREG network involving chr12 bins.

Note that the previous tables and figures include summary statistics that we computed especially for the paper, using custom Cypher and R scripts. It is not possible to generate these figures from the website.

**2. Example –Nanog’s regulatory landscape**

In this first example, we use GREG to answer five questions: (Example 2.1) Is the NANOG protein acting as a multimer?; (Example 2.2) Which TFs are regulating the Nanog gene expression?; (Example 2.3) Is the Nanog gene rich in chromatin interactions?; (Example 2.4) Is the 45-kbp-upstream-Nanog enhancer active in K562 cells?; (Example 2.5) Are there TADs around the Nanog locus?

*Example 2.1. Finding NANOG-NANOG protein interactions*

Procedure:

(a) Leave the default values in the Step 1 and Step 2 boxes.

(b) Go to the step 3 box, select “Transcription factors” and NANOG, and limit the search to chr12, between positions 7,000,000 and 7,800,000.

(c) Press “Display graph”.


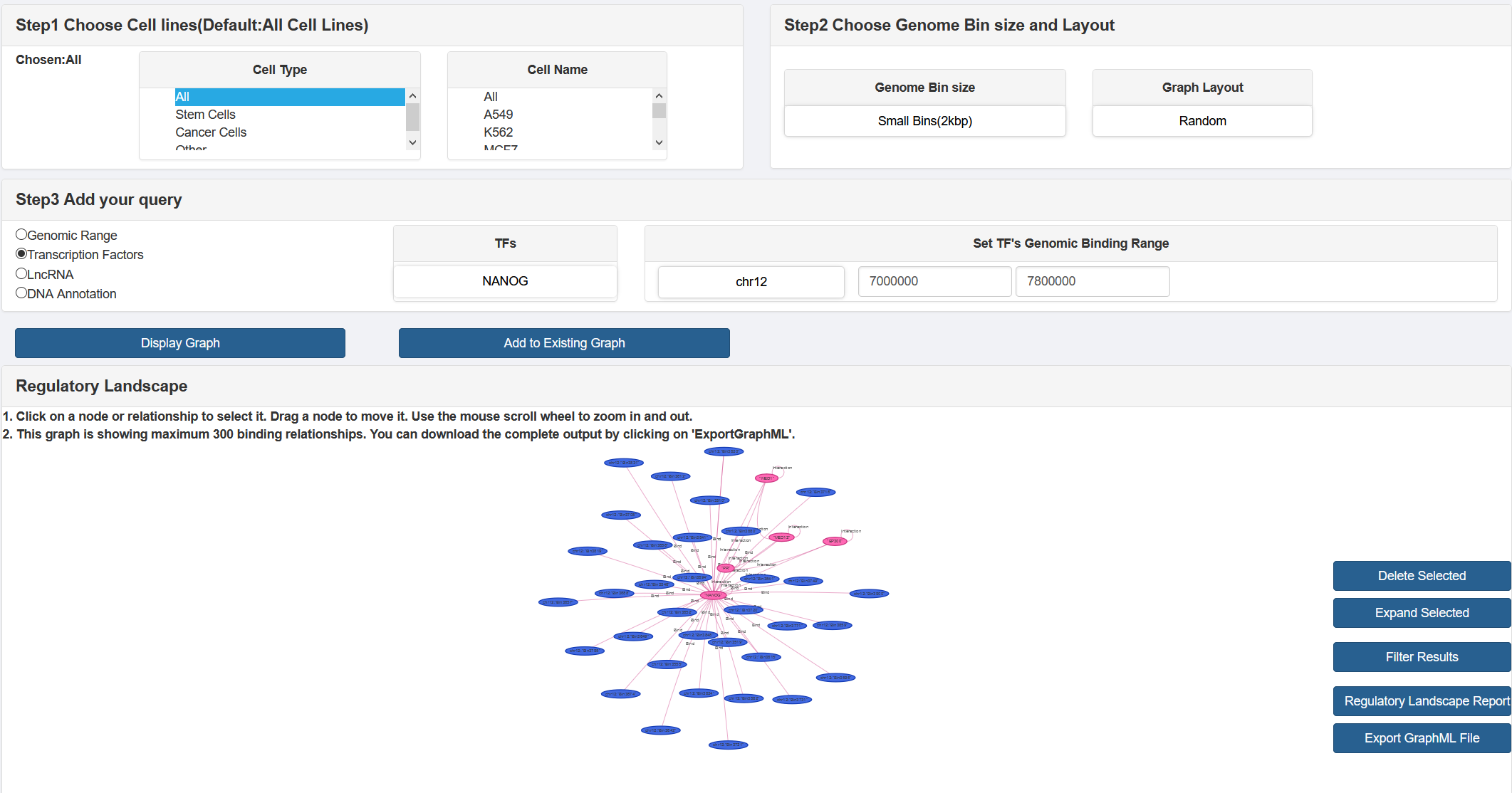


(d) Press “Filter results” and choose filter by “TF-TF Interactions”.


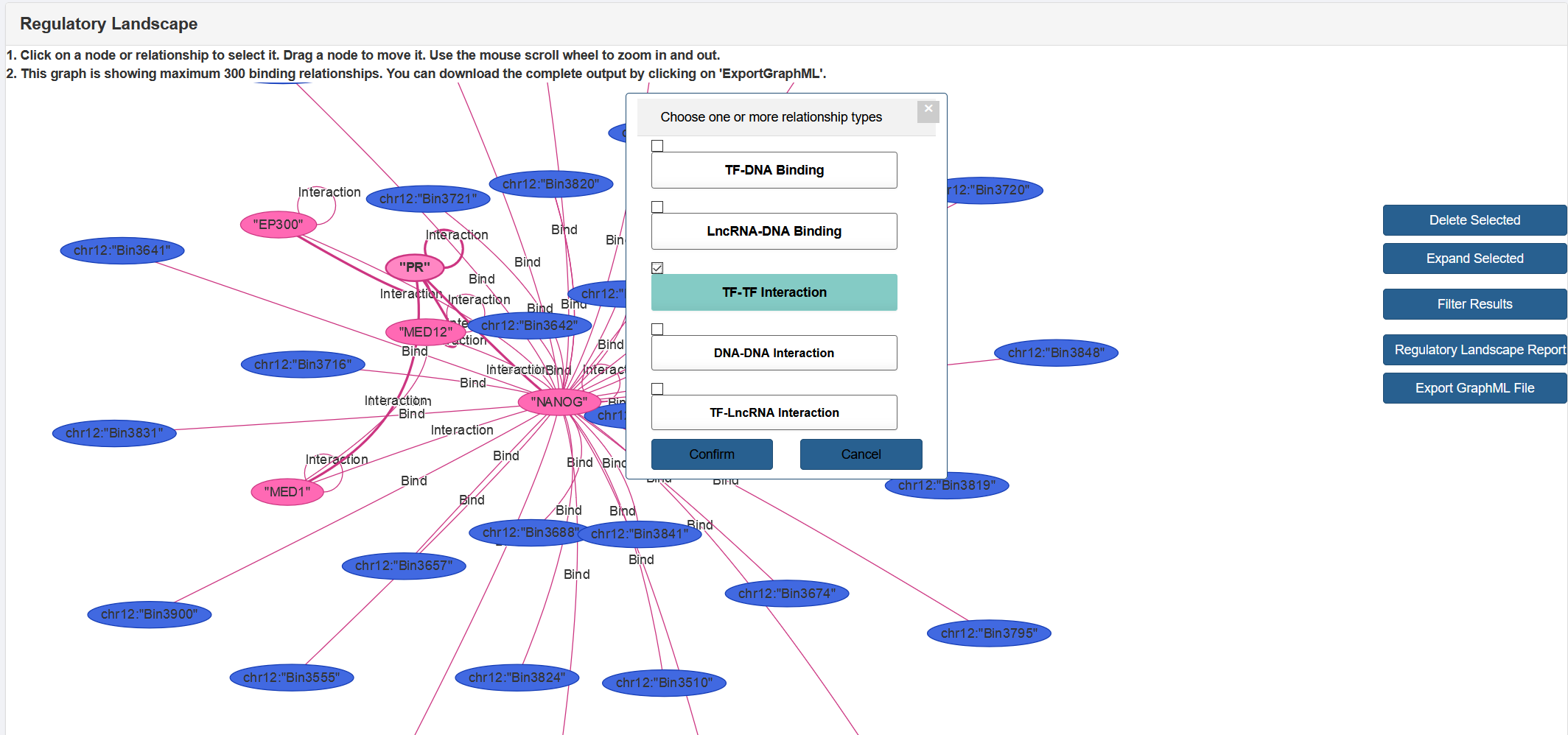


Results:


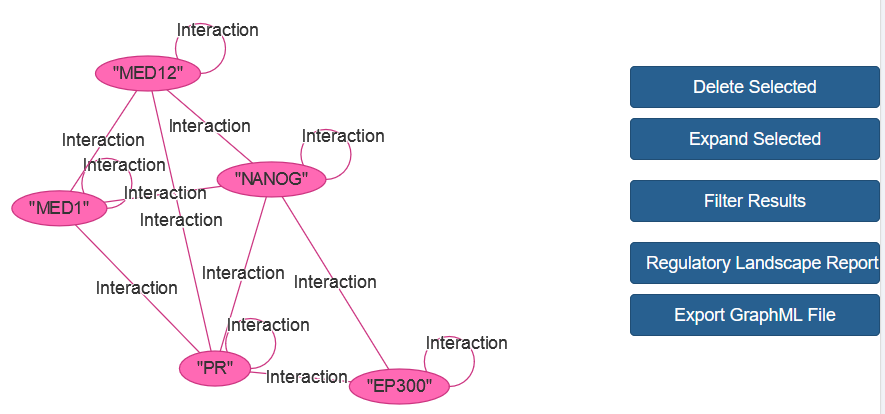


*Example 2.2. Finding TF-Nanog binding relationships*

Procedure:

(a) Get Nanog’s genomic coordinates for hg38. From GeneCards, we get: chr12:7,787,794-7,799,141

(b) Leave the default values for steps 1 and 2, and select “Genomic range” in step 3.


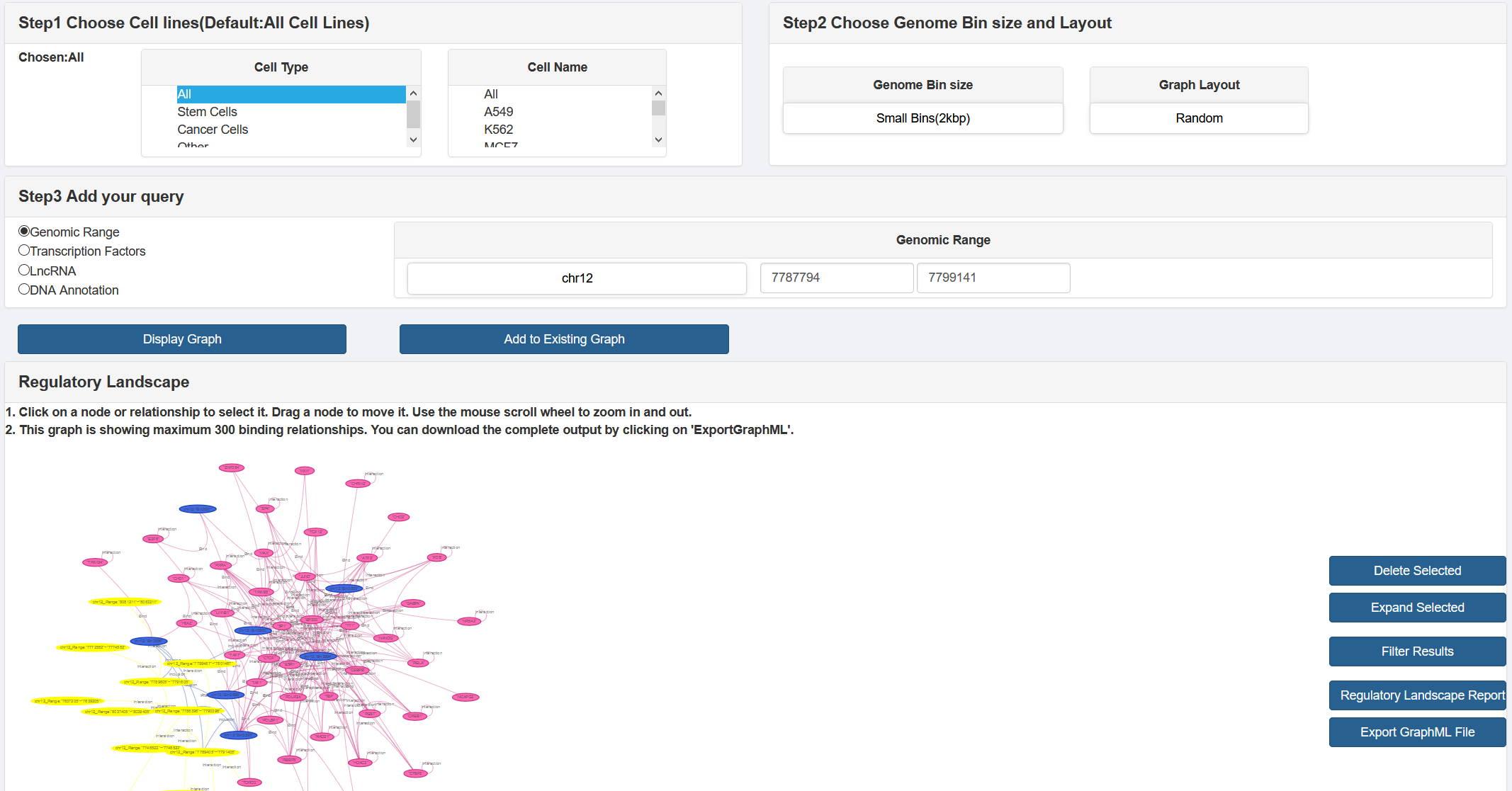
(c) The chromosome should be chr12, the lower boundary of the genomic region should be “7,787,794” and the upper boundary should be “7,799,141”. Press “Display graph”.

(d) Press “Regulatory landscape report”.


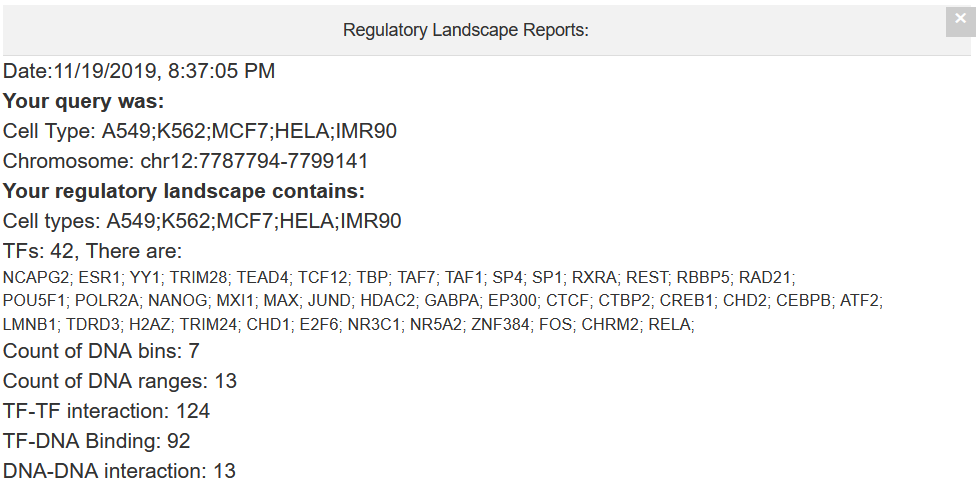


*Example 2.3. Finding DNA-DNA interactions involving Nanog*

Procedure:

(a) Leave the default values for steps 1 and 2.

(b) Go to step 3, select “DNA annotation”, and type “NANOG”.

(c) Specify chromosome 12, in the range between 7,700,000 and 7,800,000 bp. “Display graph”.


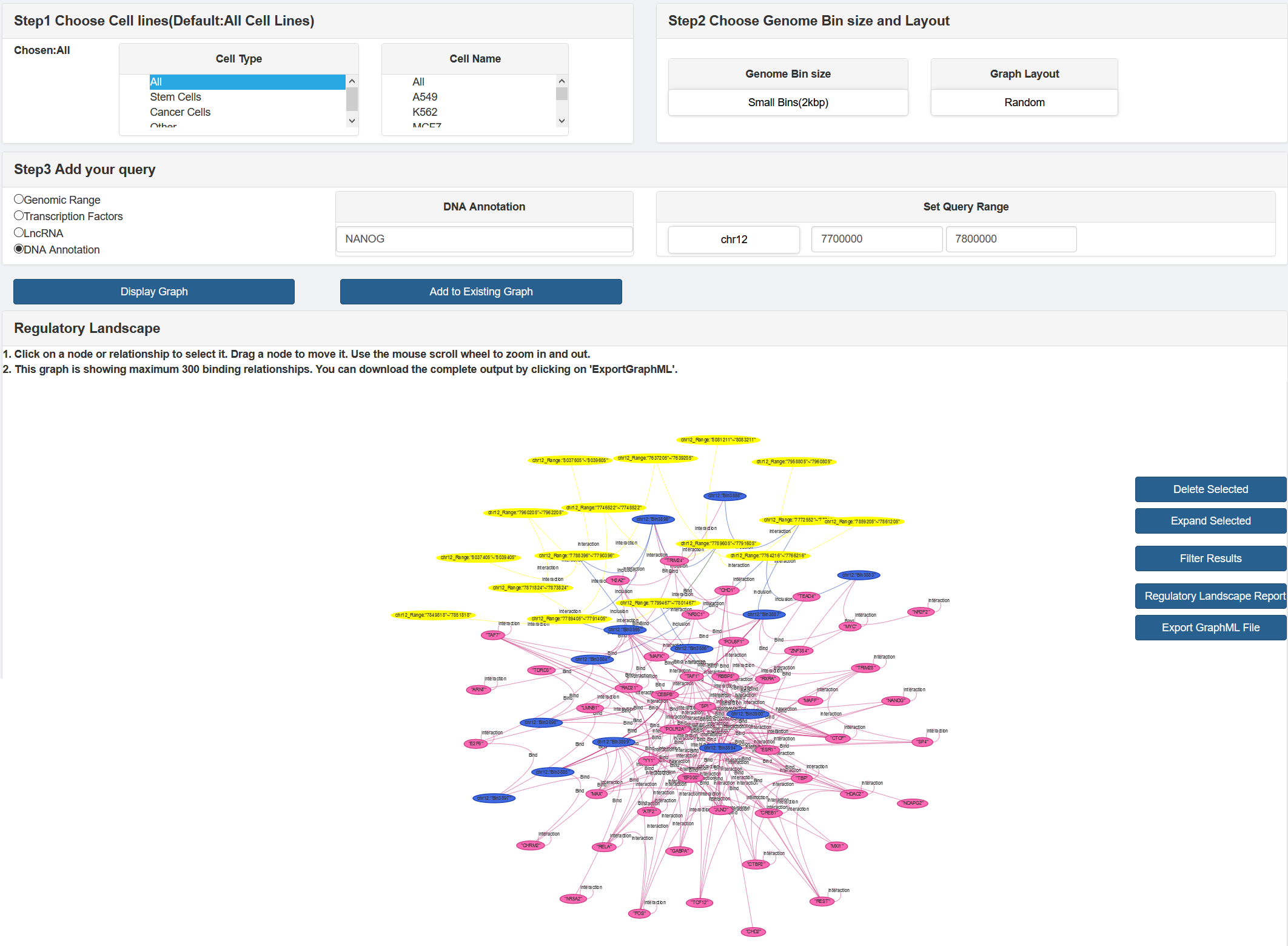


(d) Filter by DNA-DNA interactions. Press “Regulatory landscape report”.


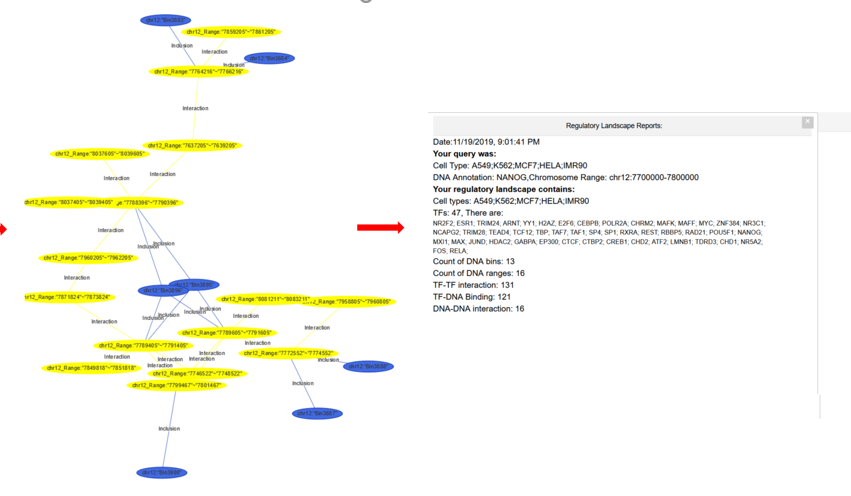


*Example 2.4. Finding interactions between Nanog and the region 45 kbp upstream Nanog*

Procedure:

(a) Choose K562 cells and leave default values in step 2.

(b) Set “DNA annotation” to “NANOG”, chr12, and coordinates from 50 kbp upstream Nanog to the end of the gene, that is, 7,737,794-7,799,141

(c) “Display graph”

(d) Filter by DNA-DNA interactions


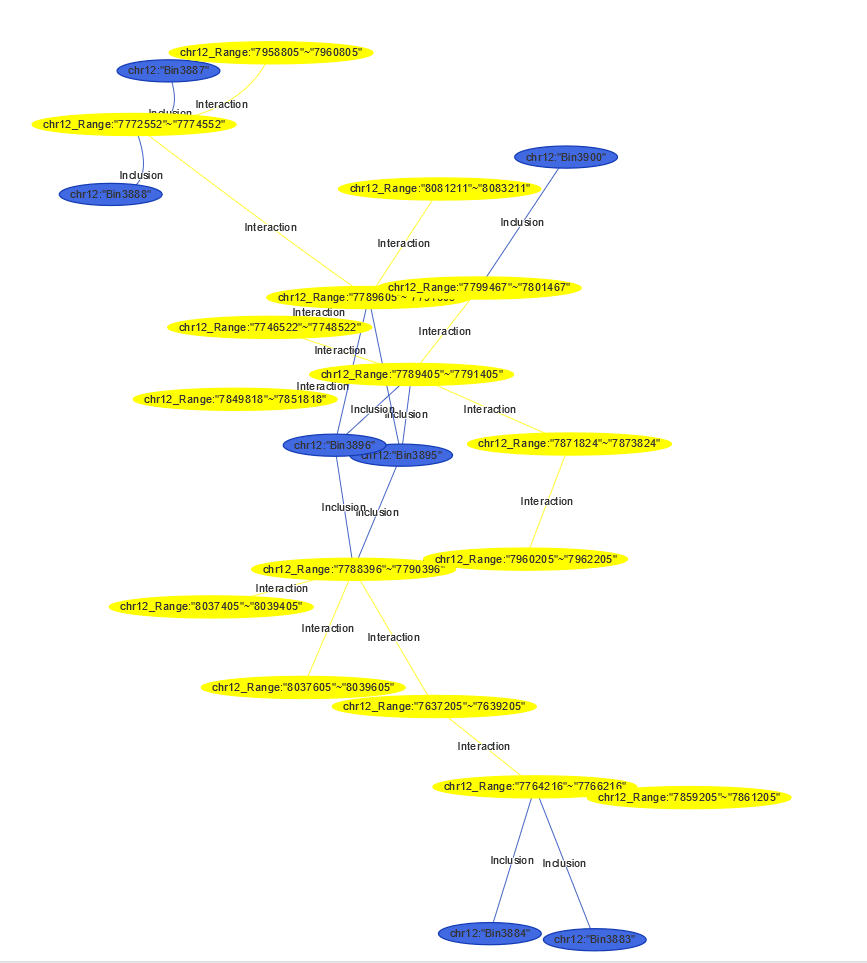


*Example 2.5. Describing TADs around Nanog*

Procedure:

(a) We obtain a list of TADs for K562 cells (<https://www.ncbi.nlm.nih.gov/geo/query/acc.cgi?acc=GSE63525>) and convert them to hg38 using UCSC’s liftOver tool (<http://genome.ucsc.edu/cgi-bin/hgLiftOver>).

(b) We find a TAD around Nanog’s coordinates. There is a TAD at chr12:7,187,404-7,647,404

(c) In step 1, select “K562”. In step 3, select “Genomic range”, add chr12, 7,187,404 and 7,647,404

(d) “Display graph” and “Regulatory landscape report”.


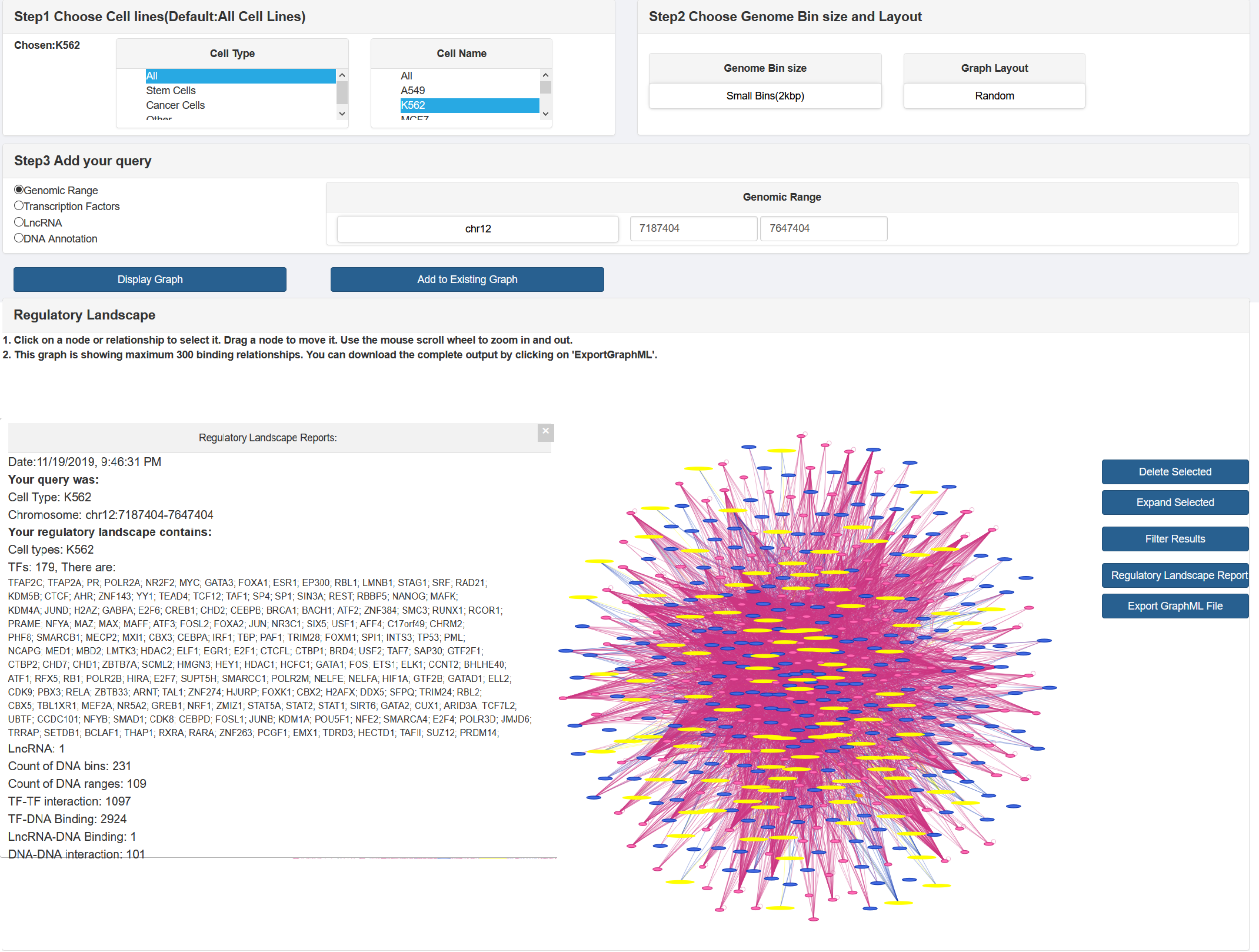


**3. Example –Exploring genomic mechanisms of COPD**

*3.1. Description of COPD candidate genes*

Table S3 summarizes basic information regarding the main COPD candidate genes. Table S4 lists the COPD-associated SNPs according to SNPedia:

**Table S3.** COPD candidate genes.

| **Gene symbol** | **Gene name** | **Associated functions of interest** |
| --- | --- | --- |
| CHRNA3 | Cholinergic Receptor Nicotinic Alpha 3 Subunit | Nicotinic acetylcholine receptor protein associated with smoking and susceptibility to lung cancer |
| CHRNA5 | Cholinergic Receptor Nicotinic Alpha 5 Subunit | Nicotinic acetylcholine receptor protein associated with susceptibility to lung cancer |
| HHIP | Hedgehog Interacting Protein | Related to the Hedgehog signaling pathway, which is important in lung morphogenesis and could be a common pathway between COPD and smoke-induced lung cancer; it also regulates pathways related to the ECM and lymphocyte activation |
| FAM13A | Family With Sequence Similarity 13 Member A | Activates Wnt signaling, and it is affected by Akt signaling |
| TGFB2 | Transforming Growth Factor Beta 2 | Part of the TGFB/SMAD pathway, which is common in proliferation and cancer |
| SERPINA1 | Serpin Family A Member 1 | Produces alpha-1 antitrypsin, which targets elastase. |
| IL13 | Interleukin 13 | Forms a cytokine gene cluster on chromosome 5q together with IL3, IL5, IL4, and CSF2 |
| MMP9 | Matrix Metallopeptidase 9 | Involved in the breakdown of extracellular matrix. Degrades type IV and V collagens |
| SOD3 | Superoxide Dismutase 3 | Antioxidant enzyme that may protect the lungs from oxidative stress |
| TGFB1 | Transforming Growth Factor Beta 1 | Part of the TGFB/SMAD pathway, which is common in proliferation and cancer |

**Table S4.** COPD-associated SNPs.

| **SNP ID** | **Gene symbol** | **Genomic coordinates (hg38)** |
| --- | --- | --- |
| rs1051730 | CHRNA3 | chr15:78,601,997 |
| rs7671167 | FAM13A | chr4:88,962,828 |
| rs17580 | SERPINA1 | chr14:94,380,925 |
| rs28929474 | SERPINA1 | chr14:94,378,610 |
| rs1800925 | IL13 | chr5:132,657,117 |
| rs17576 | MMP9 | chr20:46,011,586 |
| rs8192287 | SOD3 | chr4:24,794,946 |
| rs8192288 | SOD3 | chr4:24,795,056 |
| rs1800470 | TGFB1 | chr19:41,353,016 |
| rs1800469 | TGFB1 | chr19:41,354,391 |

*3.2. Results -Specialized chromatin hubs*

Table S5 summarizes all DNA-DNA interactions found by GREG between the bin containing the SNPs in table S4 and any other genes.

**Table S5.** DNA interactions where the first DNA range contains a COPD-associated SNP.

| **First interactor (gene containing COPD-associated SNP)** | **Second interactors** | **Second interactors down-regulated in COPD** |
| --- | --- | --- |
| CHRNA3 | CHRNA5,PSMA4,CHRNB4,AGPHD1 | AGPHD1 |
| FAM13A | - | - |
| SERPINA1 | - | - |
| IL13 | IL5,KIF3A,RAD50,IL4,MIR3936,SLC22A5,CCNI2,SEPT8 | CCNI2,IL5,KIF3A,MIR3936 |
| MMP9 | CD40,NCOA5,SLC12A5 | - |
| SOD3 | CCDC149,RN7SL16P,DHX15 | - |
| TGFB1 | HNRNPUL1,TMEM91,CCDC97,BCKDHA,EXOSC5,B9D2 | B9D2 |

The results in Table S5 lead to the following observations:

* CHRNA3 is a DNA hub at the chr15 for the genes CHRNA5 (which is another nicotinic acetylcholine receptor subunit, associated to smoking effects and lung cancer), PSMA4 (which is a proteasome subunit involved in cellular senescence and TCR signaling), CHRNB4 (another nicotinic acetylcholine receptor subunit), and AGPHD1/HYKK (another gene related to tobacco effects).

* IL13 is a DNA hub at the chr5, where interacts with cytokine genes IL5 and IL4 (related to the cytokine signaling in immune system pathway), KIF3A, SLC22A5, and SEPT8 (the first one related to microtubules and vesicle-mediated transport, the second one a transporter protein involved in elimination of toxins, and the third one related to cytoskeletal organization and cell cycle), RAD50 (related to DNA break repair and cellular senescence), and CCNI2 (a cyclin that has been associated to ashtma), as well as miRNA 3936.

* MMP9 is a DNA hub at the chr20, where interacts with the genes CD40 (antigen receptor from the TNF family, which mediates immune and inflammatory responses), NCOA5 (a nuclear co-activator and co-repressor), and SLC12A5 (a K-Cl transporter protein).

* In contrast, SOD3 and TGFB1 are DNA hubs in chr4 and chr19 respectively, where they interact with some genes poorly annotated or not related to known COPD pathways. Regions around the SNPs in FAM13A and SERPINA1 do not show chromatin interactions with other genes.

*3.3. Results -Binding of lamina associated proteins*

**Table S6.** Nuclear proteins interacting with DNA bins containing a COPD-associated SNP.

| **Gene with a COPD-associated SNP** | **Binding nuclear proteins** |
| --- | --- |
| CHRNA3 | - |
| FAM13A | RB1, LMNB1, CEBPB |
| SERPINA1 | LMNB1 |
| IL13 | CEBPB, CTCF, H2AZ, LMNB1, MAZ, MECP2, POLR2A, RAD21, RBL1 |
| MMP9 | CTCF |
| SOD3 | CEBPB, E2F7, EP300, H2AZ, LMNB1, MAZ, MECP2, MXI1, POLR2A, POLR2B, RB1, RBL1, RCOR1 |
| TGFB1 | TP53, RAD21, POLR2B, POLR2A, MXI1, MECP2, MAZ, LMNB1, H2AZ, EP300, CTCF, CHD1 |
